# Does perception of female cues modulate male short‐term fitness components in *Drosophila melanogaster*?

**DOI:** 10.1002/ece3.9287

**Published:** 2022-09-13

**Authors:** Quentin Corbel, Claudia Londoño‐Nieto, Pau Carazo

**Affiliations:** ^1^ Ethology Lab, Ethology, Ecology and Evolution Group, Cavanilles Institute of Biodiversity and Evolutionary Biology University of Valencia Valencia Spain

**Keywords:** phenotypic plasticity, reproductive behavior, reproductive plasticity, sensory perception, sexual selection

## Abstract

Phenotypic plasticity in reproductive behavior can be a strong driver of individual fitness. In species with high intra‐sexual competition, changes in socio‐sexual context can trigger quick adaptive plastic responses in males. In particular, a recent study in the vinegar fly (*Drosophila melanogaster*) shows that males derive net fitness benefits from being shortly exposed to female cues ahead of access to mating (termed *sexual perception*), but the underlying mechanisms of this phenomenon remain unknown. Here, we investigated the short‐term effects of female perception on male pre‐ and post‐copulatory components of reproductive performance: (a) mating success, (b) mating latency and duration, (c) sperm competitiveness, and (d) ejaculate effects on female receptivity and reproductive rate. We found that brief sexual perception increased mating duration, but had no effect on the other main pre‐ and post‐copulatory fitness proxies recorded. This suggests that perception of female cues may not yield net fitness benefits for males in the short‐term, but we discuss alternative explanations and future avenues of research.

## Graphical abstract

A recent study in the vinegar fly (*Drosophila melanogaster*) shows that males respond adaptively to perception of female cues in a way that increases their reproductive success, but we ignore the underlying mechanisms of this phenomenon. We found that brief sexual perception increased mating duration, but had no effect on the main pre‐ or post‐copulatory fitness proxies. These results tie up with previous findings to suggest that previously documented male adaptive responses to sexual perception may not be due to a short‐term advantage, but rather to fitness benefits that play out across the entire male lifespan.

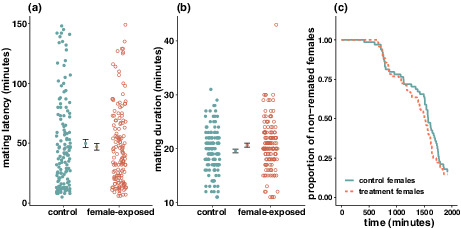

## 1 INTRODUCTION

Phenotypic plasticity is defined as the capacity of a genotype to produce alternative phenotypes depending on the environmental context it is exposed to (Bradshaw, 1965; Gause, 1947; Levins, 1963). In particular, adaptive phenotypic plasticity allows organisms to adjust their phenotype in order to cope with contrasting environmental conditions (Demmig‐Adams et al., 2008); whether adaptive phenotypic plasticity can evolve or not depends on its associated costs, and is contingent on a certain degree of environmental predictability (Botero et al., 2015; DeWitt et al., 1998; Reed et al., 2010). More specifically, plasticity in reproductive behavior (*reproductive plasticity*) is a central component of individual fitness in the face of high heterogeneity in socio‐sexual contexts (Dewsbury, 1982; Gage, 1995; Kokko & Rankin, 2006; Rebar et al., 2019). For this reason, reproductive plasticity is often considered to be a key determinant of population responses to rapid environmental change (Agrawal, 2001; Charmantier et al., 2008).

Across the animal kingdom, males tend to experience high variability in their reproductive success (Bateman, 1948; Janicke et al., 2016) and hence they may be expected to display high plasticity in their reproductive behavior in response to environmental changes (Bretman et al., 2011). For example, male plastic responses to cues reflecting intra‐sexual competition are rather well documented across distant taxa (Aragón, 2009; Bretman et al., 2011; delBarco‐Trillo & Ferkin, 2004). Males of different species have been shown to strategically adjust mating duration (Bretman et al., 2009; Sakaluk & Müller, 2008), mate guarding behavior (Carazo et al., 2007), sperm transfer (Gage, 1991; Gage & Baker, 1991), and even seminal fluid protein transfer (Wigby et al., 2009) in response to cues indicating sperm competition risk and/or intensity (e.g. Shifferman, 2012). Ultimately, high intra‐sexual competition can even lead to the evolution of alternative reproductive tactics and/or strategies (Hurtado‐Gonzales & Uy, 2010; Oliveira et al., 2008).

A recent empirical study reported that, in *Drosophila melanogaster*, short‐term exposure to female cues ahead of access to reproduction (termed *sexual perception*) can increase male relative lifetime reproductive success in a competitive environment, whereas extended exposure decreases it (Corbel et al., 2022). This plastic response seems to be adaptive across a wide range of socio‐sexual scenarios, and may explain previously documented survival and reproductive costs linked with male perception of female cues (García‐Roa et al., 2018; Gendron et al., 2014; Harvanek et al., 2017). Specifically, this study found that benefits of short exposure to female cues ahead of mating appeared to be rapidly induced (as early as over the first 24 h following access to females), and spanned across the whole lifespan of males. This is especially interesting because male responses to female cues appear to contrast from male responses to rivals in terms of effective duration; while sexual perception leads to plastic effects that accumulate along life (Corbel et al., 2022), male responses to rivals yield significant early‐life fitness benefits that rapidly disappear (Bretman, Westmancoat, Gage et al., 2013). Given that male plastic responses to sexual perception can magnify the opportunity for selection (Carazo et al., 2017; Corbel et al., 2022; García‐Roa et al., 2018) and may help explain aging in response to sensory stimuli (Gendron et al., 2014; Harvanek et al., 2017), identifying the mechanisms responsible for such male plasticity could provide valuable information.

Here, we aimed to further understand the processes leading to male sexual perception benefits by investigating the fitness consequences of perceiving female cues. To this aim, we studied the immediate effects of short‐term exposure to female cues ahead of mating on pre‐ and post‐copulatory fitness components in *D. melanogaster* males. Similarly to other polygamous species with high intra‐sexual competition, pre‐copulatory fitness of males of this species is modulated by male–male competition and female choice, both of which contribute to determine male mating success in a competitive scenario (Andersson, 1994; Arbuthnott et al., 2017; Dow & Von Schilcher, 1975). Post‐copulatory male fitness is largely driven by sperm competitiveness, which mostly depends on sperm offense in this species (see Fricke et al., 2010; Simmons & Fitzpatrick, 2012). Additionally, male manipulation of female reproductive behavior via the transfer of accessory gland proteins within the seminal fluid is known to benefit male post‐copulatory fertilization success (Aigaki et al., 1991; Chapman, 2001; Chapman et al., 2003; Chen et al., 1988; Fiumera et al., 2005; Fricke et al., 2009; Hopkins et al., 2019; Liu & Kubli, 2003; Ravi Ram & Wolfner, 2007). In fact, there is firm evidence showing that males strategically adjust the transfer of seminal fluid proteins in response to the socio‐sexual context they experience (Hopkins et al., 2019; Sirot et al., 2011; Wigby et al., 2009). Thus, in order to fully capture the effects of sexual perception on male short‐term fitness, we exposed virgin males to female cues for a period of 24 h and subsequently measured the following male fitness components: (a) mating success, (b) mating latency and duration, (c) sperm competitiveness, and (d) ejaculate effects on female receptivity and reproductive rate. We predicted that, if immediate responses are relevant to the previously documented long‐term fitness benefits of male plastic responses to female cues, exposure to female cues should improve pre‐ and/or post‐copulatory performance of males.

## 2 METHODS

### 2.1 Fly husbandry and collection

In this experiment, we used laboratory adapted *Drosophila melanogaster* wild‐type (*wt*) Dahomey flies as focal males and sparkling poliert *(spa*) mutant females and male competitors. This allowed us to discriminate the paternity‐share of focal *wt* males; the *spa* allele being recessive, individuals homozygous for this locus display the *spa* eye phenotype, whereas heterozygous *wt*/spa individuals display the *wt* phenotype. We kept stock populations at 24°C on a 12 h light/12 h dark cycle, with overlapping generations, and fed them with standard food weekly (solidified aqueous mix containing 60 g L^−1^ corn flour, 50 g L^−1^ white sugar, 40 g L^−1^ fresh baker's yeast, 10 g L^−1^ soy flour, 10 g L^−1^ industrial agar, 3 g L^−1^ Methyl 4‐hydroxybenzoate, 10 ml L^−1^ 96% EtOH, 5 ml L^−1^ 99% propionic acid). We collected eggs directly from stock populations using yeasted grape juice agar plates (FlyStuff grape agar premix, Genesee Scientific). We ensured a controlled density of ca. 200 larvae per 250 ml bottle filled with ca. 75 ml of standard food. Using ice anesthesia, we isolated virgin flies by sex 6 h upon emergence. We kept females in groups of 15 per vial and males in groups of 20 per vial. All vials used in this experiment contained a large amount of the same food the populations were fed with, both for adult feeding purposes and to provide an adequate egg‐laying substrate to females.

### 2.2 Experimental design

#### 2.2.1 Sensory treatment

We first exposed 3‐day‐old wildtype (*wt*) virgin males to females cues for 24 h. To do so, we isolated standard males in a vial, and this vial was connected to either (a) another vial containing three 3‐day‐old virgin *wt* females (i.e. female‐exposed male) or (b) to an empty vial (control male). Importantly, interconnected vials were separated by a fine mesh partition, and this allowed exchange of female semio‐chemicals (volatiles but also probably non‐volatiles) as well as female visual cues across the chambers, while ensuring males would not mate (García‐Roa et al., 2018). Previous empirical research has determined that this methodology does not elicit any courtship behavior in female‐exposed males (Corbel et al., 2022).

#### 2.2.2 Mating success

Immediately following short‐term (24 h) exposure of experimental males to female cues (or control), we discarded all donor females, and we set up 317 triplets consisting of: a female‐exposed *wt* male, a control *wt* male and a standard *wt* female. All three individuals were 4‐day‐old virgins at the start of the mating trials. To distinguish between female‐exposed and control males, we marked both males with a dot of acrylic paint on the backside of their thorax using either of two easily discernible colors (Vallejo acrylic studio; cadmium red hue N°2 “PCKPCQL” or primary blue N°24 “PBN4CQK”). We haphazardly alternated assignment of color to either treatment in order to balance any potential color‐induced bias in the behavior of reproducing females and/or focal males. We used systematic scan sampling to record male mating success (which of the two males mated with the female, if any), mating latency (time between the start of mating trials and the beginning of a successful copulation), as well as mating duration (length of a successful copulation by either male). We only considered a mating as successful if it lasted longer than 10 min (unpublished results show that this provides a conservative threshold for successful mating in this population). We ensured a one‐minute resolution in the measurement of these variables by limiting the number of vials each of the two observers handled at the time. We conducted observations following a blind protocol. After a successful mating, we discarded both males, but kept mated females alone in the vial for later experiments (see below). We gave females a total of 150 min to mate with either of the two males, after which we discarded all females that had not mated; a large proportion of the females mated with either of the two males (ca. 86%; 273 successful matings were recorded, out of 317 triplets set). Females that mated with a female‐exposed male are hereafter called *treatment females*, whereas females that mated with a control male are called *control females*.

#### 2.2.3 Post‐copulatory fitness‐ mating effects on female receptivity and productivity

To test whether males exposed to female cues could derive fitness benefits through altered female remating behavior (mediated by the differential transfer of seminal fluid proteins from the accessory glands during mating; Hopkins et al., 2019), we monogamously housed 135 focal females (71 treatment females and 64 control females) with a standard 3‐day‐old virgin male, and monitored remating latency over a period of 8 h. This was done on the day following the initial mating. We discarded successfully remated females (i.e. at least 10 min long copulation) and isolated females that had failed to remate. The next day, we presented these un‐remated females to another standard virgin male, in a new vial, for up 8 h. We ran remating trials for 4 successive days (i.e. starting 24 h, 48 h, 72 h and 96 h after the end of the first mating), after which a large proportion of the females had remated (110 out of 135, over 4 days). Females that did not remate following these 4 days were discarded, but accounted for in the remating latency analysis (right‐censored, see below). When calculating remating latency over many days, the time between two remating trials was not included; i.e. maximum remating latency over 4 days was therefore 8 h * 4 remating trials = 32 h (1920 min).

In order to assess whether exposure to female cues could lead to increased female immediate reproductive output, we also monitored the daily reproductive output of the 135 remaining females (71 treatment females and 64 control females) over the 7 days following the initial mating. Following the initial mating, we individually flipped females into a new vial every day in order to obtain a daily measure of female early‐life reproductive output. We incubated vacant vials for 15 days to allow F1 offspring emergence (average generation time being ca. 10 days), after which we froze them at ca. −20°C for later counting.

#### 2.2.4 Post‐copulatory fitness – sperm competition

**FIGURE A1:**
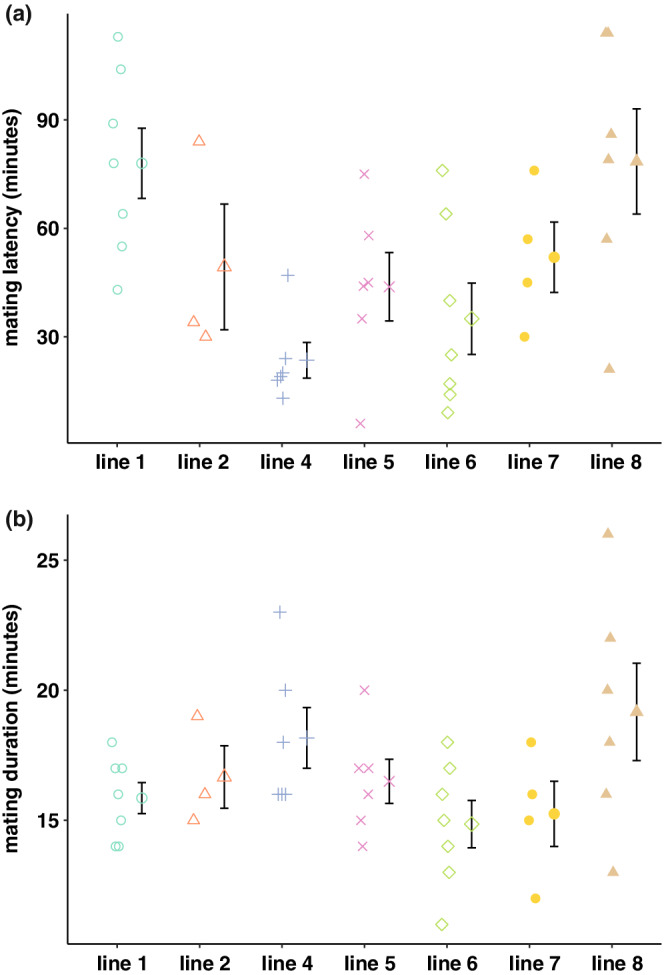
(a) Mating latency and (b) mating duration of spa males originating from 7 genetically uniform lines, in a monogamous setting with a stock spa female. Group means are displayed inwards relative to single observations, and overlapping vertical bars represent one standard error around this mean. The same number of monogamous matings were set up for each genetically uniform line (10); number of single observation thus indicates mating success of males from each line within 2 h. Line 4 was selected to provide standard sperm donors for sperm competition assays.

Ahead of sperm competition assays, we created 13 *spa* inbred lines in order to obtain genetically uniform males to compete against our focal males. We did this by mating full‐sibling *spa* originating from our stock population for three successive generations. We then selected the inbred line with the lowest inter‐individual variance in reproductive behavior (mating latency and mating duration; in a monogamous setting), with an average trait value most similar to the ancestral *spa* population, and with the strongest competitive abilities (i.e. low mating latency and high mating duration; Figure A1a,b). We then examined the effect of perception of female cues on sperm‐offense abilities (paternity share of a male mating second with a female; *P2*), as it is the main sperm competition measure explaining male fitness in *D. melanogaster* (Fricke et al., 2010). With this intent, we monogamously housed ca. 700 *spa* virgin females from stock populations with a genetically uniform *spa* virgin male for 150 min, in order for the couple to mate. After 150 min, we discarded *spa* males and kept females alone in the vial for 48 h in order to provide a realistic time lag between the two matings (i.e. similar to what *D. melanogaster* may experience in the wild; Dukas, 2020; Giardina et al., 2017; Gromko & Markow, 1993; Harshman & Clark, 1998; Imhof et al., 1998; Jones & Clark, 2003; Soto‐Yéber et al., 2018). Additionally, this 48 h time lag permitted us to adequately assess whether *spa* females successfully mated with the *spa* male, via observation of eggs/first instar larvae in the egg laying substrate. We discarded all females that did not produce at least one egg from the pool of standard mated females used for sperm competition assays, which left us with 645 mated *spa* females. Following these 48 h, we haphazardly set up 321 females to mate with control males and 324 females to mate with female‐exposed males, in fresh vials. Due to logistic limitations, we did this in two batches in which we balanced the number of replicates of each treatment (female‐exposed and control males). We recorded mating latency, mating duration and only considered a mating as successful if it lasted longer than 10 min. Following a successful mating, males were immediately discarded to prevent remating, and females were left alone in the vial. Remating trials lasted 150 min, after which we discarded all females that did not remate.

A total of 282 females remated with the male they were offered (136 female‐exposed and 146 control males). We allowed isolated females to lay eggs for 4 days, during which we flipped them into fresh yeasted vials every day. We then incubated vials for 15 days to allow F1 offspring emergence (average generation time being ca. 10 days). We then froze them at ca. ‐20°C for later counting of offspring of each phenotype (*wt* or *spa*). We pooled the offspring count from the four consecutive days in order to score sperm‐offense abilities of the focal male. We discarded females that did not produce a single viable offspring during these 4 days (seven females) from further analyses, as no focal male paternity share could be computed. We also discarded two females from further analyses due to human error (one female escaped and one was erroneously discarded). Our final sample size was 273 (*n* = 132 treatment females, *n* = 141 control females).

We computed sperm offense (P2) as the proportion of offspring sired by the focal (*wt*) male:

$$P2=\frac{N_{\mathrm{wt}}\;}{N_{\mathrm{spa}}+N_{\mathrm{wt}}}$$

Where *N*_*wt*_ is the absolute number of offspring sired by the focal (*wt*) male, and *N*_*spa*_ is the absolute number of offspring sired by the standard competitor (*spa*) male.

In this species, virgin females are relatively unselective, and mating increases female choosiness (Bateman, 1948; Kohlmeier et al., 2021). As a consequence, we also extracted data on male mating success, mating latency and mating duration from the P2 assays. The rationale was to gauge male plastic reproductive behavior when presented to a choosier female, and thus in a context where inter‐sexual competition is expected to be more important.

### 2.3 Statistical analyses

We analyzed differences in mating success between female‐exposed and control males using a one‐sample Wilcoxon signed rank test with continuity correction; each female was assigned a binary response representing mate choice (“0” for control male, “1” for female‐exposed male), with mu set at 0.5 as females should have no preference for either of the two males under H_0_. We analyzed mating latency and mating duration of female‐exposed versus control males using Kruskal‐Wallis rank sum tests in which treatment (female‐exposed vs control) was the sole categorical predictor. We analyzed remating latency of treatment vs control females using a Cox proportional hazard model (Cox, 1972) with treatment as predictor, and we right‐censored females that did not remate after the 4 days. We graphically and statistically verified that the assumptions of the Cox proportional hazard model were met using Schoenfeld residuals diagnostics (Schoenfeld, 1982). We analyzed female early‐life reproductive success (daily offspring production over 7 days) using a general linear mixed model (“lme4” R package; Bates et al., 2015) with treatment, day and their interaction as categorical fixed effects, and female ID as random effect. We extracted the absolute values of the residuals‐vs‐fitted from an initial heteroskedastic model and used them as weights in order to meet the homoskedasticity assumption of the linear model (Midi et al., 2009, 2013).

We analyzed mating success of female‐exposed and control males during the P2 assays (monogamously presented to mated females) by fitting a generalized linear mixed model with a binomial error distribution (“0” if male failed to mate, “1” if male succeeded), and with treatment as fixed effect predictor and batch as random effect. We analyzed mating latency of female‐exposed vs control males when monogamously presented to mated females using a Cox proportional hazard model (Cox, 1972) with treatment as predictor. We right‐censored males that did not mate after the 150 min. We analyzed mating duration of female‐exposed vs control males when monogamously presented to a mated female by fitting a general linear mixed model with treatment as fixed effect predictor and batch as random effect. Finally, we analyzed sperm‐offense data in a generalized linear mixed model with a beta‐binomial error distribution using the “glmmTMB” R package (Brooks et al., 2017). We transformed this data in order to meet the beta distribution range (i.e. *y*′ = (y*[*N* − 1] + 0.5)/*N*; Smithson & Verkuilen, 2006). Treatment was the sole fixed effect predictor included in this model, and batch was the only random effect.

We ran all statistical tests in R version 4.0.3 (R Core Team, 2020). For all tests, we set *α* = 0.05, ran type III ANOVA and checked model assumptions using the “performance” R package (Lüdecke et al., 2021). We corrected for multiple comparison using the Benjamini and Hochberg (1995) step‐up procedure for a false discovery rate of 0.05. Outcome of this procedure is detailed when relevant (potential cases of false positive) in the result and discussion sections. We produced all figures using the “ggplot2” R package (Wickham, 2016).

## 3 RESULTS

**FIGURE 1:**
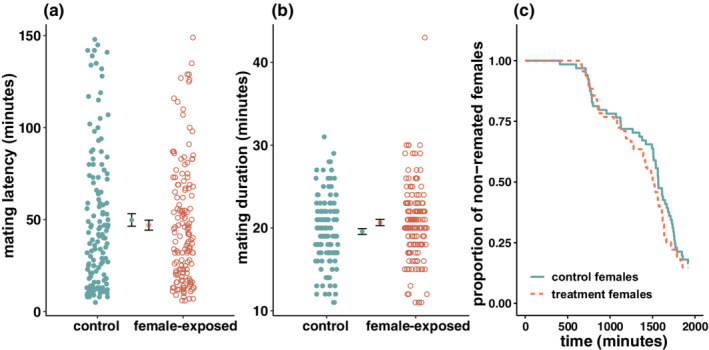
(a) Mating latency and (b) mating duration of control males (green filled circles) and female‐exposed males (orange empty circles) in reciprocal contest environment. Group means are displayed inwards relative to single observations, and overlapping vertical bars represent one standard error around this mean. (c) Females remating latency following a single mating with either a control male (solid green line) or a female‐exposed male (dashed orange line). Females were given 8 h to mate every day for 4 consecutive days following initial mating (summing up to 32 h of remating trials).

**FIGURE 2:**
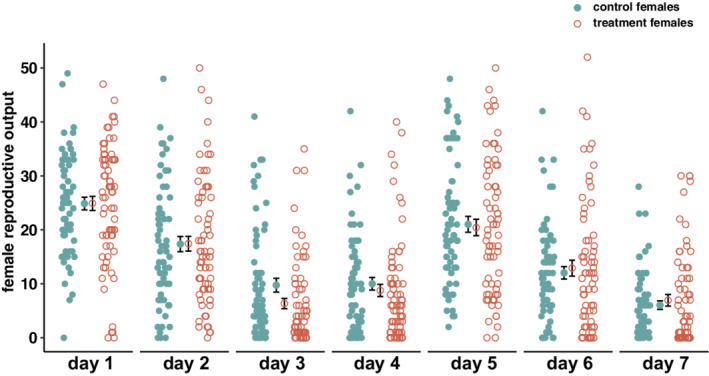
Daily reproductive output of control females (green filled circles) and treatment females (orange empty circles). Group means are displayed inwards relative to single observations, and overlapping vertical bars represent one standard error around this mean. Treatment females are females that initially mated with a 24 h female‐exposed male, whereas control females are females that initially mated with a control male (isolated for 24 h).

Around 52% of the standard virgin females simultaneously presented to a female‐exposed and a control male mated with the female‐exposed male (143 out of 273 realized matings), with no evidence that exposure to female cues significantly affected mating success (i.e. deviation from the expected 50%; *V* = 19,800, *p* = .398). We did not observe a significant difference in mating latency between female‐exposed and control males (K‐W *χ* ^2^ < 0.001, *p* = .995; Figure 1a). We found that exposure to female cues significantly increased mating duration (K‐W *χ* ^2^ = 4.523, *p* = .033; Figure 1b), however the Benjamini and Hochberg (1995) procedure for multiple testing correction indicated that this result may represent a false discovery, given our significance threshold set at *α* = 0.05. Average remating latency did not differ significantly between treatment females and control females (K‐W *χ* ^2^ = 0.719, *p* = .397; Figure 1c). We found no significant effect of mating with either a female‐exposed or a control male on female early‐life reproductive success ($\chi_{1}^{2}$ = 0.155, *p* = .694; Figure 2), and this was consistent across days ($\chi_{1}^{2}$ = 7.290, *p* = .295; Figure 2). However, we found a significant effect of day ($\chi_{1}^{2}$ = 164.359, *p* < .001; Figure 2).

**FIGURE 3:**
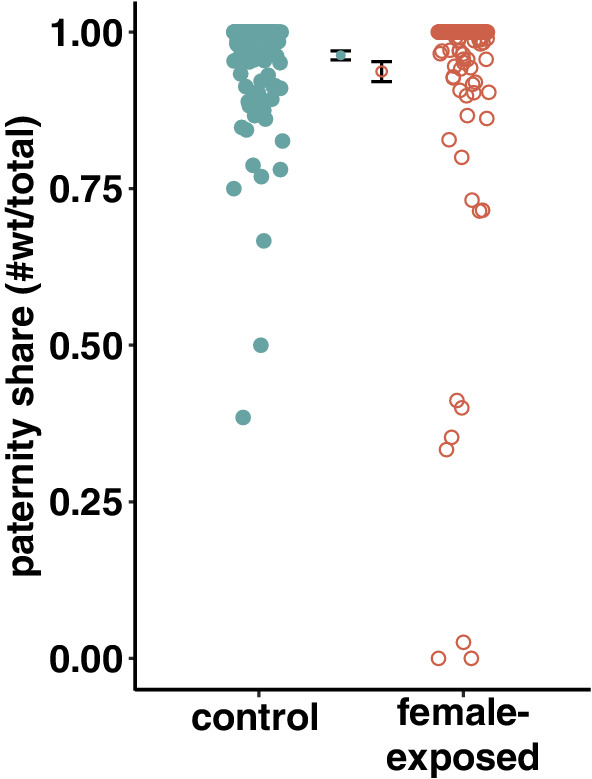
Sperm offense abilities of control males (green filled circles) and female‐exposed males (orange empty circles). Sperm offense is calculated at the proportion of offspring sired by a focal male mating with a previously mated female. Group means are displayed inwards relative to single observations, and overlapping vertical bars represent one standard error around this mean.

**FIGURE A2:**
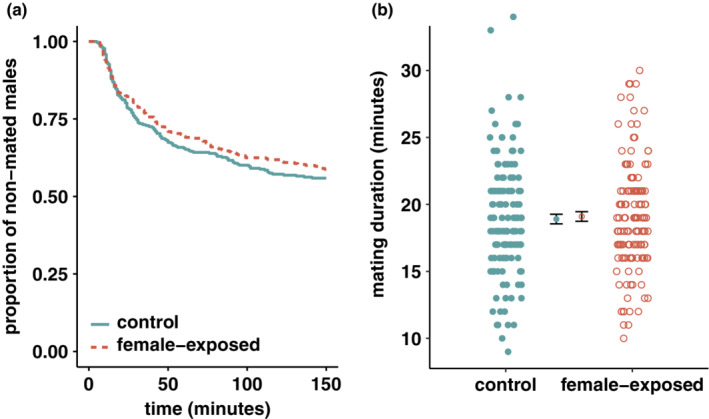
(a) Mating latency of control male (solid green line) or a female‐exposed male (dashed orange line) when monogamously housed with a previously mated female. Males that did not mate following the 150 minutes of mating trial were right‐censored in the statistical analysis. (b) Mating duration of control males (green filled circles) and female‐exposed (orange empty circles) when monogamously housed with a previously mated female. Group means are displayed inwards relative to single observations, and overlapping vertical bars represent one standard error around this mean.

We found no significant differences in mating success ($\chi_{1}^{2}$ = 0.843, *p* = .359), mating latency ($\chi_{1}^{2}$ = 0.918, *p* = .338, Figure A2a) or mating duration ($\chi_{1}^{2}$ = 0.1213, *p* = .7276, Figure A2b) between female‐exposed and control males monogamously presented to mated females (P2 experiment). We found no significant difference in sperm competitiveness (i.e. sperm offense, *P2*) between female‐exposed and control males (*χ* ^2^ = 0.015, *p* = .902; Figure 3).

## 4 DISCUSSION

Overall, we found no conclusive evidence that the lifetime fitness benefits of sexual perception previously reported (Corbel et al., 2022) are due to immediate effects on male fitness components. We found no differences in mating success or mating latency between control males and female‐exposed males (Figure 1a), showing that males do not seem to derive pre‐copulatory benefits from short‐term exposure to female cues. In our assays, focal males competed directly against each other over a female. Thus, these results reflect the net outcome of intra‐ and inter‐sexual pre‐copulatory competition in a biologically relevant scenario (Dukas, 2020). The use of virgin females may have reduced our ability to detect differences in male inter‐sexual competitiveness given that, in this species, virgin females are considerably less choosy than mated females (Bateman, 1948; Kohlmeier et al., 2021). However, data from sperm competition assays shows that female‐exposed males do not seem to differ from control males in either mating success or mating latency when exposed to mated (and thus choosier) females (Figure A2a).

We did find evidence that short‐term exposure to female cues resulted in significantly increased mating duration (Figure 1b), such that female‐exposed males mated on average for 1′05″ longer than control males (i.e. a 5.54% increase). In *D. melanogaster*, mating duration is mainly driven by males (Bretman, Westmancoat, & Chapman, 2013; MacBean & Parsons, 1967), and longer matings often translate into higher reproductive success for males (Bretman et al., 2009; Wigby et al., 2009). Thus, albeit small in magnitude, this difference could be biologically meaningful. However, this difference was flagged as a potential false discovery when correcting for inflation of experiment‐wise type I error rate (i.e. Benjamini & Hochberg, 1995), and should hence be interpreted with caution. We found that female‐exposed males did not increase mating duration when copulating with previously mated females (in P2 assays; Figure A2b). Empirical evidence shows that, in *D. melanogaster*, sperm transfer is completed before the midpoint of copulation (Manier et al., 2010), and that longer matings do not yield higher sperm transfer (Gilchrist & Partridge, 1997, 2000). In fact, previous research has linked the fitness benefits associated with longer matings to the transfer of non‐sperm components that increase immediate oviposition rate in females (see Chapman et al., 2003; Chapman & Davies, 2004; Wigby et al., 2009). However, we found no significant differences in daily reproductive output of females over the 7 days following mating with female‐exposed vs control males (Figure 2). In promiscuous species, female remating rate is often under high sexual conflict. While males benefit from females not remating (to avoid post‐copulatory competition), females can benefit from remating with several males (e.g. through increased offspring genetic diversity; Yasui, 1998; Arnqvist & Rowe, 2005). In this context, males of this species have evolved seminal fluid proteins that allow them to decrease female receptivity following mating (Perry et al., 2013). We found no difference in remating latency between treatment females and control females (Figure 1c), suggesting that female‐exposed males are not more successful at manipulating female receptivity than control males. It should be noted that *D. melanogaster* males are known to adjust their ejaculate size depending on female mating status (Lüpold et al., 2011; Sirot et al., 2011); future research could investigate the effects of sexual perception on the ejaculate content of female‐exposed vs control males using mated females. Altogether, we did not find support that the increase in mating duration of males presented to virgin females yields post‐copulatory fitness benefits. This suggests that, within the limitations of our experimental design, this increase was not biologically relevant for the post‐copulatory fitness proxies investigated, or that it is a false discovery.

In polygamous species with high mating rates, sperm competitiveness can be an important driver of individual fitness (Firman & Simmons, 2011; Schnakenberg et al., 2012; Singh et al., 2002). Sperm competitiveness is often measured as the paternity share that a male achieves when competing against another male within the female reproductive tract. The paternity share of the first of two males to mate with the female will define his *sperm defense* abilities, whereas the paternity share of the second male will define his *sperm offense* abilities (Boorman & Parker, 1976). In *D. melanogaster*, sperm‐offense abilities correlate more strongly with relative lifetime reproductive success than sperm‐defense abilities (Fricke et al., 2010). We thus investigated whether exposure to female cues ahead of mating could affect sperm‐offense abilities, and found no significant difference between female‐exposed males and control males (Figure 3). It is worth noting that, given the relatively high baseline level of paternity share of control males (ca. 95.17% ± 0.85 SEM), our study may lack power to pick up any effects of exposure to female cues on *P2*. The fact that we used *sparkling* males as rival males in this remating assay may contribute to this high level, as *spa* males are poorer competitors than *wt* males. Thus, cryptic female choice in favor of focal males may obscure any difference between treatments. This being said, P2 levels above 90% are common in *Drosophila melanogaster*, across experiments using *spa* competitors (Fiumera et al., 2005) or not (Patlar & Civetta, 2022; Yeh et al., 2012). Therefore our result does not appear to deviate much from what is expected for this species.

In conclusion, we explored an array of pre‐ and post‐copulatory short‐term male fitness components and found no clear indication that any of the components measured are affected by brief sexual perception. While this means that the mechanisms leading to enhanced reproductive performance of males following sexual perception are still unidentified, our results suggest that whatever these mechanisms are, they do not result in an improvement of males' immediate fitness. This, in turn, would support the previously mentioned idea that sexual perception benefits build up along the span of males' lifes (several weeks after initial perception of female cues) to yield a net lifetime fitness gain (Corbel et al., 2022).

## AUTHOR CONTRIBUTIONS

**Quentin Corbel:** Conceptualization (equal); data curation (lead); formal analysis (lead); investigation (lead); methodology (equal); visualization (equal); writing – original draft (equal); writing – review and editing (equal). **Claudia Londoño‐Nieto:** Investigation (supporting); methodology (supporting); writing – review and editing (supporting). **Pau Carazo:** Conceptualization (equal); funding acquisition (lead); investigation (supporting); methodology (equal); project administration (lead); resources (lead); supervision (lead); writing – original draft (equal); writing – review and editing (equal).

## FUNDING INFORMATION

Q.C. was supported by a Generación del Conocimiento research grant PID2020‐118027GB‐I00 to P.C. P.C. was funded by MCIN/AEI/10.13039/501100011033. C.L.N. was supported by a MINECO predoctoral grant from the Spanish Government (PRE2018‐084009) to P.C.

## CONFLICT OF INTERESTS

Authors declare no conflicting interests.

### OPEN RESEARCH BADGES

This article has earned Open Data, Open Materials and Preregistered Research Design badges. Data, materials and the preregistered design and analysis plan are available at https://doi.org/10.5061/dryad.dfn2z354d.

## Data Availability

Data and code have been deposited in the Dryad Digital Repository (https://doi.org/10.5061/dryad.dfn2z354d).
